# Innovative Advances in Non-Invasive Detection Technologies for Heart Failure: Synergistic Application of Multimodal Sensing and Intelligent Algorithms 

**DOI:** 10.31083/RCM48196

**Published:** 2026-06-24

**Authors:** Yixin Chen, Qiyao Yu, Hongfei Xu, Lingxian Zhang, Weidong Li, Yongbing Wu

**Affiliations:** ^1^Queen Mary College, Jiangxi Medical College, Nanchang University, 330006 Nanchang, Jiangxi, China; ^2^Department of Cardiothoracic Surgery, The Second Affiliated Hospital, Jiangxi Medical College, Nanchang University, 330006 Nanchang, Jiangxi, China; ^3^Department of Cardiovascular Surgery, The First Affiliated Hospital of Zhejiang University, School of Medicine, 310003 Hangzhou, Zhejiang, China

**Keywords:** heart failure, non-invasive monitoring, artificial intelligence, machine learning, wearable electronic devices, telemedicine, precision medicine

## Abstract

Heart failure (HF) remains a leading global cause of chronic disease-related disability and mortality, with rising incidence driven largely by population aging. Early diagnosis is challenging because initial symptoms are often subtle and non-specific, leading to delayed detection and poor prognosis. While conventional tools such as echocardiography and B-type natriuretic peptide (BNP) testing remain diagnostic gold standards, these approaches are limited by operator dependency, restricted accessibility, and dynamic monitoring. Recent advances in artificial intelligence (AI) and cloud computing have enabled a new generation of non-invasive, intelligent technologies that integrate wearable sensors (*e*.*g*., ReDS™) with multimodal platforms (*e*.*g*., HeartLogic™, CardioSignal) to support real-time risk tracking and personalized management. Indeed, supported by favorable policy environments and strengthened collaboration among manufacturers, clinicians, and researchers across multiple fields and disciplines, the development of intelligent non-invasive HF detection devices has accelerated, leading to rapid innovation, commercialization, and continuous emergence of novel technologies and products. This review systematically summarizes HF pathophysiological mechanisms and current clinical monitoring strategies. Moreover, this review critically evaluates emerging devices and AI-driven platforms, highlighting the associated underlying principles, data integration capabilities, and clinical applicability. Finally, the analysis addresses key challenges, including the “black box” dilemma associated with AI, data bias, and privacy concerns, and proposes future directions for early screening, risk stratification, and precision intervention. By synthesizing technological comparisons and limitations, this review aims to provide a comprehensive reference for advancing intelligent HF diagnostics.

## 1. Introduction

Heart failure (HF) represents a major public health challenge and remains one of the leading causes of morbidity and mortality worldwide. As a worldwide epidemic, HF currently affects over 64 million people, with an estimated prevalence of around 2% among adults [1]. This prevalence is expected to continue rising due to the aging population. Demographic shifts, including aging populations and improved survival among individuals with cardiovascular risk factors, are expected to further increase its prevalence. Around 80% of HF cases are concentrated in low- and middle-income countries, highlighting significant disparities in healthcare resources and preventive strategies [2].

The prognosis of HF remains grave, with five-year mortality rates exceeding 50%, underscoring the substantial clinical and socioeconomic burden it imposes. In the United States, the prevalence of HF is projected to increase by 46% between 2012 and 2030, accompanied by an alarming 127% rise in associated healthcare costs [3]. In China, the age-standardized prevalence of HF among adults aged 25 and above is 1.10%, while the age-standardized prevalence among those aged 35 and above is 1.38% [4].

**Fig. 1.**
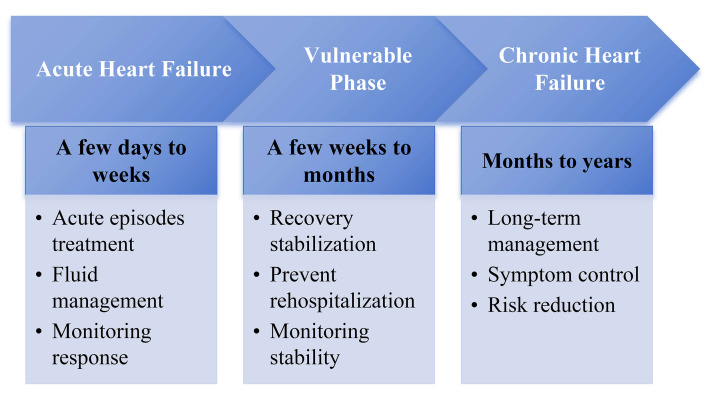
**Clinical staging and evolution pathway of heart failure (HF)**. Core Management Goals during Acute Decompensation, Vulnerable, and Chronic Stable Phases.

Acute decompensation can occur rapidly and unpredictably, whether in chronic HF or newly diagnosed HF [5,6] (Fig. 1). Recent HF guidelines highlight the importance of early diagnosis and immediate treatment for acute heart failure (AHF) patients to reduce disease progression and improve outcomes [5]. “Time is prognosis” is particularly relevant in HF management [7]. However, current diagnostic modalities, such as echocardiography and measurements of B-type natriuretic peptide (BNP) or N-terminal pro-BNP (NT-proBNP), face several limitations, including operator dependency, limited accessibility in primary care settings, and an inability to provide real-time monitoring. These limitations limit their utility in early warning systems and continuous disease management. Furthermore, certain hemodynamic assessments still rely on invasive methods such as pulmonary artery catheterization, which is complex and costly, with risks of complications such as infections and bleeding. This makes it difficult to widely implement in routine follow-up and community-based care, especially in the post-pandemic era, when patients demand more home-based healthcare solutions [8].

Therefore, there is an urgent need to develop novel non-invasive diagnostic strategies that can accurately identify patients with HF both during hospitalization and after discharge, enabling comprehensive and real-time risk assessment to improve prognostic outcomes. Non-invasive portable devices allow continuous measurement of physiological parameters. Their portability and ease of use are expected to enhance patient engagement in disease management, potentially reducing outpatient visits and hospital readmissions, thereby alleviating burdens on healthcare systems and optimizing resource allocation [9,10,11].

Advances in “AI + medical devices” are paving the way for intelligent data-processing platforms that integrate remote monitoring (RM), multi-parameter fusion models, and AI-assisted diagnostics. These innovations significantly enhance the diagnostic validity of collected data, transforming non-invasive HF detection devices into integrated platforms capable of “detection, analysis, diagnosis, and management”, supporting more precise diagnostics and personalized treatment strategies [12,13].

This review summarizes the underlying principles of non-invasive intelligent HF detection, surveys current and emerging technologies in portable non-invasive devices, and examines the integration of artificial intelligence (AI) and big data analytics in this domain, both domestically and internationally. Furthermore, it discusses the current and anticipated clinical value of these innovations in improving HF outcomes.

## 2. Literature Review

### 2.1 Pathophysiology and Clinical Features of Heart Failure

HF is a complex clinical syndrome resulting from structural or functional cardiac abnormalities, which impair ventricular ejection or filling capacity. In clinical practice, commonly used classification systems include symptom-oriented grading, disease progression staging, and ejection fraction-based classification, each of which provides a comprehensive assessment framework from the perspectives of functional status, disease stages, and underlying pathological mechanisms [14,15,16]. The New York Heart Association (NYHA) functional classification system categorizes HF into grades I to IV based on patients’ exercise tolerance and symptom severity, which is widely used for clinical evaluation and prognosis. The American College of Cardiology/American Heart Association (ACC/AHA) staging system, ranging from Stage A to Stage D, emphasizes the dynamic progression of HF, from the high-risk group in Stage A to end-stage HF in Stage D, highlighting the clinical value of early recognition and intervention. This model highlights the importance of early intervention to delay or prevent disease progression. The left ventricular ejection fraction (LVEF)-based classification has gained prominence in defining HF phenotypes and guiding therapeutic decisions. It divides HF into three subtypes: heart failure with reduced ejection fraction (HFrEF, LVEF ≤40%), heart failure with mildly reduced ejection fraction (HFmrEF, LVEF 41–49%), and heart failure with preserved ejection fraction (HFpEF, LVEF ≥50%). These subtypes exhibit distinct pathophysiological features, clinical presentations, and responses to treatment [14,17,18]. In China, HF guidelines align with international standards, integrating the above three classification systems as a core framework. Additional tools, such as the Killip classification for acute myocardial infarction-related HF, as well as objective assessments, such as the six-minute walk test, are employed for quantitative functional evaluation, enabling stratified and personalized management [19].

The core mechanisms of HF can be summarized as sustained overactivation of neurohormonal systems, hemodynamic disturbances, and dysregulation of myocardial energy metabolism. These three mechanisms are intimately interconnected: hemodynamic alterations often serve as the initial trigger, neurohormonal overactivation acts as a persistent amplifier driving disease progression, and impaired energy metabolism represents a fundamental intrinsic factor leading to functional decompensation. Together, they form a self-perpetuating vicious cycle that promotes both the development and gradual worsening of HF.

#### 2.1.1 Hemodynamic Derangements

Hemodynamic derangements constitute a pivotal component in the pathophysiology of HF, exerting profound effects on vital organs such as the lungs and kidneys.

In the setting of HF, alterations in cardiac output (CO) combined with elevated cardiac filling pressures give rise to a cascade of hemodynamic abnormalities. In the kidney, elevated central venous pressure (CVP) leads to renal venous congestion, which has been strongly linked to progressive renal dysfunction as well as resistance to diuretic therapy. In the lungs, elevation of the pulmonary capillary wedge pressure (PCWP) precipitates pulmonary congestion and increased capillary wall stress, ultimately leading to pulmonary vascular remodeling and structural injury [20,21]. Collectively, these hemodynamic perturbations play a critical role in shaping disease progression and long-term prognosis.

From a clinical standpoint, right heart catheterization (RHC) remains the gold standard for obtaining hemodynamic parameters, thereby offering essential insights that inform both the diagnosis and the therapeutic management of HF [22,23].

#### 2.1.2 Neurohormonal Hypothesis

Persistent activation of neurohormonal pathways is widely recognized as a fundamental driver in the onset and progression of HF. This concept was first articulated by Packer [24] and colleagues in the form of the “neurohormonal hypothesis”, which proposed that HF is not merely the consequence of hemodynamic abnormalities, but is predominantly driven by excessive activation of endogenous neurohormonal systems, most notably the sympathetic nervous system (SNS) and the renin–angiotensin–aldosterone system (RAAS).

Sustained elevations of norepinephrine and angiotensin II directly induce cardiomyocyte apoptosis, fibrosis, and downregulation of β-adrenergic receptors, ultimately leading to irreversible deterioration in cardiac structure and function. Beyond these direct myocardial effects, increased neurohormonal activity also promotes secondary maladaptation such as fluid retention, peripheral vasoconstriction, and arrhythmogenesis, thereby perpetuating a vicious cycle of disease progression [25].

These neurohormonal perturbations are reflected by a spectrum of measurable biomarkers, including plasma catecholamines, norepinephrine, and endothelin, which serve as minimally invasive indicators of HF status. Importantly, neurohormone-related biomarkers not only mirror disease severity but also provide valuable insights into therapeutic response and prognostic risk, thus supporting precision monitoring and individualized intervention strategies [25,26,27].

#### 2.1.3 Energy Metabolic Dysfunction

Disruption of cardiac energy metabolism represents another key mechanism underlying the complex pathophysiology of HF. Under physiological conditions, the healthy myocardium derives its energy primarily from fatty acid and glucose oxidation. However, in HF, profound metabolic remodeling occurs, leading to an “energy-starved” state characterized by depleted phosphocreatine (PCr) stores, reduced ATP availability, and impaired energy transfer systems [28,29,30].

A hallmark of this reprogramming is the shift in substrate preference. In many forms of HF, there is downregulation of fatty acid oxidation (FAO) pathways, accompanied by a compensatory increase in glucose utilization. This metabolic shift is particularly pronounced in HFpEF. Although the decrease in FAO has been interpreted as a potential adaptive response, this altered metabolic pattern fails to adequately compensate for impaired glucose metabolism. Ultimately, these metabolic disturbances lead to a critical energy deficit, worsened by mechanical and energy uncoupling and insufficient reserve during stress [28,29].

### 2.2 Clinical Detection Indicators for Heart Failure

#### 2.2.1 Clinical Symptoms and Signs

Traditionally, the diagnosis of HF has largely relied on clinical presentation and physical examination. Key manifestations include dyspnea reflecting elevated pulmonary pressures, peripheral edema and fluid retention associated with right ventricular dysfunction, fatigue and reduced exercise tolerance due to diminished CO and impaired skeletal muscle perfusion, as well as cough and wheezing secondary to pulmonary congestion and bronchial edema. However, reliance on symptoms and signs is inherently subjective, highly variable among individuals, and limited by poor specificity and sensitivity. Additionally, accurate interpretation often depends on the clinician’s experience, which may lead to inconsistencies and diagnostic inaccuracies. Moreover, conventional symptomatology offers no capacity for continuous or dynamic monitoring of a patient’s hemodynamic status or disease trajectory, limiting the real-time assessment of treatment response or early detection of decompensation [18,31,32].

As a result, while clinical evaluation remains a foundational aspect of HF diagnosis and monitoring, there is a growing emphasis on integrating objective biomarkers and imaging modalities to enhance accuracy, support phenotypic stratification, and enable personalized management.

#### 2.2.2 Cardiac Imaging Techniques

Cardiac imaging plays a pivotal role in the diagnosis and evaluation of HF. Commonly used modalities include cardiac magnetic resonance imaging (CMR), three-dimensional echocardiography (3D Echo), and cardiac computed tomography (CT). These techniques are complementary, allowing for comprehensive evaluation of cardiac structure and function, identification of underlying pathologies, and guidance for personalized treatment strategies [33,34].

Despite their diagnostic value, each modality presents significant limitations that constrain widespread clinical applicability. CMR and CT are costly, resource-intensive, and have restricted availability. The procedures are time-consuming and require specialized operators and interpretation. Additionally, CT carries the risk of ionizing radiation exposure, while contrast agents may induce renal impairment or allergic reactions. Echocardiography, while widely available, remains highly dependent on operator skill and is susceptible to image degradation due to adverse patient characteristics, such as obesity or pulmonary disease, which may introduce diagnostic subjectivity. Moreover, most imaging modalities provide intermittent and static assessments, capturing anatomic and functional data at a single time point. This inherent limitation inhibits continuous hemodynamic monitoring and thus impedes the timely detection of dynamic changes in HF status [34,35].

#### 2.2.3 Biomarkers

Biomarkers are of critical value in the diagnosis, phenotyping, disease monitoring, and prognostic evaluation of HF. BNP and NT-proBNP are the most widely used markers, whose circulating levels rise in response to increased ventricular wall stress and volume overload, supporting early diagnosis, the assessment of therapeutic interventions, and prognostication. Cardiac troponins (cTnI/cTnT), core diagnostic markers of acute myocardial infarction, when persistently elevated, indicate ongoing myocardial injury and are strongly associated with adverse outcomes. D-dimer, in addition to reflecting thrombogenesis, may signal hemodynamic abnormalities and disease severity in HF patients [36,37,38,39].

Despite their clinical utility, biomarkers are constrained by several limitations. Natriuretic peptides are influenced by renal function, with chronic kidney disease often producing falsely elevated results due to impaired clearance, thus reducing diagnostic specificity [40,41]. Pharmacologic agents also complicate interpretation. For instance, angiotensin receptor–neprilysin inhibitor (ARNI) can artifactually raise BNP concentrations [42,43]. Furthermore, conditions such as atrial fibrillation (AF), obesity, and advanced age may attenuate the diagnostic accuracy of NT-proBNP, particularly in HFpEF [44]. Elevated troponin levels, while indicative of myocardial injury, may also result from non-cardiac conditions such as diabetes, chronic kidney disease, or left ventricular hypertrophy, thereby increasing false-positive results [45]. D-dimer is elevated in diverse settings such as infection, thrombosis, and systemic inflammation, limiting its specificity for HF. Although markers of renal, hepatic, and electrolyte imbalance provide insights into multi-organ involvement in HF, they often exhibit low sensitivity and specificity, typically becoming abnormal only in advanced disease stages. In addition, repeated blood sampling imposes a significant burden on patients and healthcare systems, potentially compromising long-term adherence to monitoring protocols.

#### 2.2.4 Genomic and Metabolomic Analyses

Molecular profiling provides further insights into HF pathogenesis and management. Transcriptomic analysis of blood or myocardial tissue enables the identification of gene expression signatures associated with myocardial remodeling, inflammatory activation, and metabolic dysregulation, providing a molecular basis for early detection, prognostic stratification, and individualized treatment strategies [46,47]. Metabolomic profiling, on the other hand, offers a systems-level view of biochemical alterations in HF by quantifying small-molecule metabolites in biofluids or tissues. These metabolic biomarkers not only increase the understanding of HF pathophysiology but also hold potential for early diagnosis, disease classification, and personalized therapeutic strategies [48,49,50].

Despite these advances, the translation of omic technologies into routine clinical practice remains challenging. Issues such as standardization of analytical protocols, computational complexity in data integration, high costs of specialized instrumentation, and the need for specialized expertise continue to limit widespread implementation.

#### 2.2.5 Electrophysiological Monitoring

Electrophysiological abnormalities are highly prevalent in HF and serve as critical predictors of disease progression and sudden cardiac death. These abnormalities can be detected via surface or implantable electrodes during myocardial depolarization and repolarization. In conventional 12-lead electrocardiography (ECG), parameters such as QRS duration and corrected QT interval (QTc) provide vital prognostic information. QRS prolongation (≥120 ms) has been established as a critical marker, with studies demonstrating that patients with prolonged QRS exhibit significantly higher two-year all-cause mortality (59%) compared to those with normal QRS (37%), corresponding to an almost twofold increase in risk (HR = 1.94, *p* < 0.01) [51,52,53,54]. Similarly, QTc prolongation is strongly associated with cardiovascular mortality and an increased risk of 30-day mortality in patients with acute HF [55].

In addition to traditional metrics, emerging evidence highlights the electromechanical activation time (EMAT) and its heart rate–corrected value (EMATc) as important markers for HF monitoring. EMAT, defined as the interval from the Q wave onset on ECG to the first heart sound (S1) on phonocardiography, reflects the duration from electrical activation to aortic valve opening, serving as an indicator of electromechanical coupling efficiency. It correlates closely with left ventricular contractility, demonstrating a sensitivity of 96.1% and specificity of 87.0% for detecting systolic dysfunction [56]. Elevated EMAT has been identified as an independent risk factor for adverse in-hospital events, including cardiogenic shock and death, in chronic HF patients. An admission EMATc >13.8% was predictive of major adverse cardiac events (MACEs) with 81.8% sensitivity, 65.9% specificity, and an area under the curve (AUC) of 0.799 [57]. Furthermore, EMAT independently predicts cardiac mortality in out-of-hospital HF populations, highlighting its utility as a non-invasive, dynamic index for risk stratification [58].

In clinical practice, the 12-lead ECG remains the most widely used electrophysiological evaluation tool due to its non-invasiveness, low cost, and ease of use, providing rapid recordings within minutes for initial screening and acute evaluation. However, it possesses significant limitations. Standard ECG captures only a single time point, offering limited sensitivity for intermittent or subclinical arrhythmias, and its diagnostic accuracy is highly dependent on operator expertise [55]. A meta-analysis reported a median interpretation accuracy of only 54% among physicians, which improved to only 67% following structured educational interventions [59,60,61].

Holter monitoring (ambulatory ECG) extends recording periods to 24–48 hours or longer, improving the detection of paroxysmal arrhythmias during daily activities. However, its utility is constrained by bulky equipment, reduced patient comfort and compliance due to multiple leads, and potential skin irritation. Furthermore, the finite monitoring window may still miss infrequent arrhythmic events [62].

### 2.3 The Evolution and Current Status of Non-Invasive Detection of Heart Failure

Since the development of the string galvanometer by Einthoven in 1903 and its application to cardiac electrophysiology, the foundation for non-invasive monitoring of cardiac electrical activity has been firmly established. Over the past century, non-invasive detection of HF has undergone a remarkable evolution (Table 1). From the early 1900s to the mid-to-late 20th century, research efforts were largely devoted to the discovery of non-invasive HF detection technologies. From the mid-20th century to the early 21st century, investigations increasingly focused on optimizing conventional methods and expanding their clinical applications. Since the beginning of the 21st century, collaboration between researchers and industry has resulted in further advances and innovations that have enhanced device usability, sensitivity, and integration of intelligent features, thereby strengthening the competitiveness of HF non-invasive monitoring technologies (Table 1).

**Table 1.** **Landmark events in the evolution of non-invasive detection of heart failure (HF)**.

Year	Event	Significance
1903	Einthoven invented the string galvanometer and initiated the clinical application of the ECG	Established modern ECG, laying the foundation for non-invasive cardiac electrophysiological monitoring.
1947	Holter completed the first wireless ECG recording	Transitioned traditional ECG from short-term detection of cardiac activity to continuous monitoring.
1961	Invention of the Holter monitor	Significantly improved the capacity for diagnosing and risk-stratifying heart diseases.
1966	First introduction of ICG	Pioneered a non-invasive pathway for deriving hemodynamic parameters from physiological signals.
1969	Clinical application of ICG achieved	ICG became an established non-invasive tool for cardiovascular functional assessment and was later widely commercialized.
1976	Frazin et al. obtained the first human left ventricular M-mode echocardiogram via the esophagus	Marked the advent of transesophageal echocardiography, enabling non-invasive structural and hemodynamic assessment that became central to HF diagnosis and management.
1996	Prototype of OptiVol technology established	Laid the foundation for intelligent implantable devices, advancing HF management from rhythm monitoring to integrated physiological surveillance.
2003	Chronicle IHM submitted for FDA approval	First integrated remote early-warning capabilities for HF into a therapeutic device, facilitating a shift from passive treatment to proactive intervention.
2004	InSync Sentry™ CRT-D approved by FDA	First clinically approved device using intrathoracic impedance technology, incorporating remote monitoring to enable proactive HF management.
2006	Inert gas rebreathing (non-invasive cardiac output measurement) was approved by FDA	Provided a non-invasive alternative to imaging techniques for measuring cardiac output, broadening physiological assessment options.
2014	CardioMEMS implantable pulmonary artery pressure monitor approved by FDA	First FDA-approved implantable system for pulmonary artery pressure monitoring, establishing the remote “pressure-guided therapy” paradigm.
2015	ReDS™ wearable system approved by FDA	Enabled quantitative assessment of pulmonary congestion at the bedside and in home settings.
2017	HeartLogic™ multiparameter early-warning system approved by FDA	First multiparameter early-warning platform for HF, enhancing risk stratification through integrated sensor data.
2025	CardioTag three-in-one sensor approved by FDA	World’s first wearable patch synchronizing ECG, PPG, and SCG technologies, supporting multidimensional cardiovascular monitoring.

ICG, impedance cardiography; IHM, Implantable Haemodynamic Monitor; FDA, Food and Drug Administration; PPG, photoplethysmography; SCG, seismocardiography; ECG, electrocardiography.

Currently, non-invasive monitoring devices for HF are highly diverse and multifunctional. Based on their detection principles, these systems can be broadly categorized into four classes: electrophysiological signal monitoring, hemodynamic monitoring, tissue fluid status monitoring, and multi-parameter integrated monitoring. From a design perspective, devices are commonly classified as wearable, patch-based, or smartphone/external peripheral–based solutions (Table 2).

**Table 2.** **Summary of representative non-invasive heart failure (HF) detection devices and approval status**.

Product type	Product name (company, country)	Approval status	Product positioning	Technology type	Market status/stage
Wearable	Zio Patch (iRhythm Technologies, San Francisco, CA, USA)	FDA approved; CE Mark	Clinical-grade wearable patch primarily used for continuous ambulatory ECG monitoring	Single-lead wireless ECG	Mature clinical deployment
	ZOLL LifeVest (ZOLL Medical Corporation, Chelmsford, MA, USA)	FDA approved; CE Mark	Clinical wearable defibrillator and continuous rhythm monitoring device in high-risk patients	Single-lead wireless ECG	Established clinical use
	Apple Watch (Apple Inc., Cupertino, CA, USA)	FDA cleared (ECG function); CE Mark; NMPA registered	Consumer-oriented smartwatch with ECG capability, mainly used for lifestyle health monitoring	Single-lead wireless ECG	Mature global consumer market
	Huawei Watch (Huawei Technologies Co., Ltd., Shenzhen, Guangdong, China)	CE Mark; NMPA registered (ECG function)	Consumer-oriented smartwatch with ECG functionality for daily health monitoring	Single-lead wireless ECG	Mature global consumer market
	Samsung Galaxy Watch (Samsung Electronics Co., Ltd., Suwon, Gyeonggi-do, Republic of Korea)	FDA cleared (ECG function); CE Mark	Consumer-oriented smartwatch for lifestyle health monitoring	Single-lead wireless ECG	Mature global consumer market
	Fitbit Charge/Sense (Fitbit LLC [Google], San Francisco, CA, USA)	Limited regulatory clearance	Consumer health-focused wearable watch; increasingly adopted in clinical studies	PPG-based HR monitoring; ECG	Mature consumer health market
	Garmin Vivosmart/Fenix (Garmin Ltd., Olathe, KS, USA)	Not medical-device approved	Sports- and fitness-oriented wearable watch	PPG-based heart rate; pulse oximetry	Mature consumer health market
	Whoop Strap (WHOOP, Inc., Boston, MA, USA)	Not medical-device approved	Screenless wearable for continuous physiological monitoring, primarily used in fitness assessment	PPG-based heart rate, HRV	Primarily used in fitness, recovery, and wellness assessment
	Owlet Dream Sock (Owlet Baby Care Inc., Lehi, UT, USA)	FDA approved; CE Mark	Consumer-oriented infant monitoring device designed for home-based physiological surveillance	Photoplethysmography	Primarily used in fitness, recovery, and wellness assessment
	VitalPatch (VitalConnect, Inc., San Jose, CA, USA)	FDA approved; CE Mark	Clinical-grade multiparameter patch used for continuous ECG and vital sign monitoring in inpatient and post-discharge settings	Single-lead wireless ECG	Early to mid-stage clinical adoption
	FloPatch® (Flosonics Medical Inc., Sudbury, ON, Canada)	FDA approved; CE Mark	Clinical wireless Doppler ultrasound cardiac monitor	Continuous-wave Doppler ultrasound	Early clinical deployment
	Vpatch System (Vpatch Cardio Pty Ltd., Sydney, NSW, Australia)	FDA approved; CE Mark	Clinical wireless multi-lead ECG monitoring patch	3-lead wireless ECG	Regional clinical deployment
	BioBeat Monitoring Patch (BioBeat Technologies Ltd., Tel Aviv, Israel)	FDA cleared; CE Mark	Clinical-grade continuous vital sign monitoring patch primarily used in patients with hypertension	Cuffless blood pressure monitoring; PPG-based multiparameter sensing	Early clinical adoption
	ReDS™ System (Sensible Medical Innovations Ltd., Netanya, USA)	FDA approved; CE Mark	Clinical wearable monitoring system designed for non-invasive assessment of pulmonary fluid status in HF patients	Electromagnetic dielectric measurement technology	Clinical trial and early deployment stage
	Zephyr BioHarness (Zephyr Technology Corp., Annapolis, MD, USA)	Not medical-device approved	Research-use chest-worn physiological monitoring system for research and performance assessment	ECG-derived heart rate; activity sensing	Research-use device
	Oura Ring (Oura Health Ltd., Oulu, Finland)	Not medical-device approved	Consumer wearable ring for continuous health and physiological monitoring; increasingly used in cardiovascular and sleep-related research	PPG-based heart rate; SpO_2_	Mature consumer health market
	Movano Evie Ring (Movano Health Inc., Pleasanton, CA, USA)	FDA cleared	Consumer-oriented smart ring for heart rate and health monitoring	PPG; accelerometer	Early commercial deployment
Smartphone/Peripheral devices	KardiaMobile series (AliveCor, Inc., Mountain View, CA, USA)	FDA approved; CE Mark	Portable ECG device used for point-of-care and home-based rhythm assessment	Single-/multi-lead ECG	Mature point-of-care device
	Vscan Air (GE HealthCare, Chicago, IL, USA)	FDA approved; CE Mark	Wireless handheld ultrasound imaging device for bedside and outpatient cardiac imaging	Dual-probe ultrasound (with Doppler)	Established clinical expansion
	Eko CORE 500 Digital Stethoscope (Eko Health, Inc., Oakland, CA, USA)	FDA approved; CE Mark	Digital stethoscope with ECG functionality for clinical auscultation and cardiac assessment	3-lead ECG	Mature point-of-care device
	Cardiio Rhythm (Cardiio Inc., Boston, MA, USA)	Not approved	Smartphone camera-based rhythm monitoring software in research and pilot studies	Photoplethysmography	Research stage
	“Wenxin Xiaoyi” Smart Heart Patch (Wenxin Technology Co., Ltd., Beijing, China)	NMPA registered	Clinical portable phonocardiography-ECG synchronous monitor	Single-lead ECG	Hospital trial stage

FDA, U.S. Food and Drug Administration; CE Mark, Conformité Européenne; NMPA, National Medical Products Administration; HR, heart rate; HRV, heart rate variability; SpO_2_, peripheral oxygen saturation.

#### 2.3.1 Monitoring of Hemodynamic and Tissue Fluid Status

Hemodynamics represents the core parameter system for evaluating cardiovascular function, which encompasses the direct measurement of indices such as CVP, pulmonary artery pressure (PAP), and pulmonary PCWP, as well as derived variables including CO, cardiac index (CI), stroke volume (SV), and systemic vascular resistance (SVR). These indices provide refined assessments of cardiac pump performance, vascular tone, and circulating volume status. In HF management, non-invasive hemodynamic monitoring devices are critical, since they quantify cardiac efficiency and evaluate the balance between vascular resistance and tissue perfusion. By facilitating early detection of circulatory dysfunction and decompensation, these tools support timely intervention and optimization of personalized treatments.

Fluid retention and dysregulated distribution are hallmark features of HF, particularly pulmonary interstitial and pleural fluid accumulation, which are closely associated with exacerbation of symptoms and risk of rehospitalization. Tissue fluid monitoring technologies track dynamic changes in body water content and identify thoracic or systemic fluid overload, thereby offering objective data for early volume status assessment, individualized diuretic therapy, and regulation of electrolytes.

**Fig. 2.**
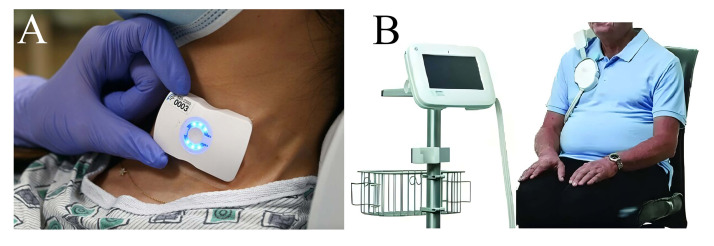
**Hemodynamic and tissue fluid status monitoring devices for heart failure (HF)**. (A) The FloPatch®, with an aperture approximately 3–4 times the width of the carotid artery, is affixed to the patient’s neck using a medical adhesive patch. The device wirelessly connects to a tablet application, enabling the acquisition of real-time hemodynamic parameters within 3 minutes. (B) The ReDS™ System utilizes a non-invasive sensing device, which is typically integrated into a wearable vest for measurement. It provides an intuitive numerical readout and trend analysis of the measured data.

Currently, representative technologies include thoracic impedance analysis (e.g., ReDS™ System) and non-invasive Doppler ultrasound devices (e.g., FloPatch®). Many of these systems incorporate wireless data transmission and intelligent analytical algorithms, enabling acquisition of real-time, non-invasive parameters and enhancing clinical decision-making through accurate, continuous monitoring (Fig. 2) (Table 3, Ref. [63,64,65,66]).

**Table 3.** **Representative intelligent monitoring devices for heart failure (HF) based on hemodynamic and tissue fluid status assessment**.

Device	Key features	Clinical evidence			Limitations
		Evidence level	Key studies	Outcomes	
Flopatch®	Wearable wireless Doppler ultrasound; non-invasive adhesive patch; real-time fluid responsiveness assessment.	Physiological validation studies; Observational clinical studies	Kenny et al., 2022 [63]; Kenny et al., 2023 [64]	Demonstrated good agreement with transesophageal echocardiography and carotid Doppler measurements for detecting stroke volume changes and hemodynamic trends [63,64].	Lack of HF-specific randomized outcome trials; limited data on long-term home use; measurement accuracy sensitive to probe positioning and user handling.
ReDS™ System	Contact-free, non-invasive measurement of lung fluid content; standing test (<90 s).	Randomized controlled trials; observational studies; meta-analyses	ReDS-SAFE HF trial; Lala et al., 2021 [66]; Alvarez-Garcia et al., 2024 [65]	Associated with reduced HF rehospitalization rates and improved post-discharge risk stratification [65,66].	Designed primarily for intermittent outpatient use; absence of standardized clinical implementation pathways; limited evidence for long-term mortality benefit.

RCTs, Randomized Controlled Trials; ReDS-SAFE HF, Use of ReDS for a SAFE discharge in patients with acute heart failure.

FloPatch®: Since its FDA clearance in 2019, FloPatch® has been recognized as the world’s first wireless wearable Doppler ultrasound device. With a compact and disposable design (135 mm × 108 mm × 43.3 mm), it utilizes ultrasound and Doppler principles to non-invasively assess blood flow. A small amount of gel is applied to the monitoring site, and the device is appropriately placed. Within seconds, key hemodynamic indices are acquired and transmitted wirelessly via Bluetooth, enabling real-time analysis and supporting timely clinical decision-making.

Validation studies demonstrated that FloPatch accurately detects SV changes when compared with transesophageal echocardiography in perioperative and critical care settings [67]. Additional feasibility studies confirmed its ability to track preload-induced hemodynamic changes and central hypovolemia [68]. While direct HF-specific outcome trials are currently lacking, extensive evidence supports the clinical value of assessing dynamic fluid responsiveness, highlighting FloPatch’s potential role in future studies of HF management.

ReDS™ System: The ReDS™ System utilizes low-power electromagnetic signals to assess dielectric properties within thoracic tissues, enabling non-invasive quantification of pulmonary fluids. This technology provides rapid measurement of lung fluid levels (expressed as a percentage) within approximately 45 seconds, offering clinicians data for timely therapeutic adjustments.

Early validation studies demonstrated a strong correlation between ReDS measurements and CT-derived lung fluid volume, supporting its physiological accuracy [69]. Subsequent prospective studies showed that residual pulmonary congestion detected by ReDS at discharge was associated with an increased risk for 30-day HF readmission [70]. In the Use of ReDS for a SAFE discharge in patients with acute Heart Failure (ReDS-SAFE HF) trial, patients managed with ReDS-guided therapy demonstrated significantly improved pulmonary congestion and a 90.6% reduction in the 30-day risk of unscheduled visits, rehospitalization, or death compared with controls [65].

However, the system’s performance may be limited in specific patient subgroups. Studies have found reduced accuracy in severely obese individuals, likely due to altered tissue dielectric properties and signal attenuation. Additionally, the presence of a sizable pleural effusion can interfere with the accuracy of measurements, as the technology primarily targets lung parenchymal fluid. These limitations raise practical concerns regarding its generalizability for long-term monitoring, particularly in home-based settings where comorbid conditions may be prevalent [71,72].

#### 2.3.2 Electrophysiological Signal Monitoring

Electrophysiological signal monitoring represents one of the most mature and widely adopted categories among non-invasive approaches for HF management. These systems capture cardiac electrical activity through surface electrodes and enable detailed analysis of rhythm disorders, conduction abnormalities, and heart rate variability. They are commonly used to detect arrhythmias, QT/QRS abnormalities, and to assess the risk of the progression of HF.

The earliest prototype of the ECG was developed by Willem Einthoven based on single-lead technology, for which he was awarded the Nobel Prize in Physiology or Medicine. Today, the 12-lead ECG has evolved into the gold standard in clinical settings. Compared with the single-lead ECG, the 12-lead system provides a more comprehensive representation of cardiac electrical activity from multiple directions, enhancing the accuracy of cardiac evaluations and supporting the diagnosis of a broad spectrum of cardiovascular diseases [73].

Although conventional Holter monitoring (24–48 hours) using skin-attached patches allows the detection of intermittent arrhythmias such as tachycardia, bradycardia, premature beats, and pauses, clinical studies have demonstrated that the median time to the first arrhythmic event is 3–4 days, exceeding the detection window of standard Holter monitoring [62].

**Fig. 3.**
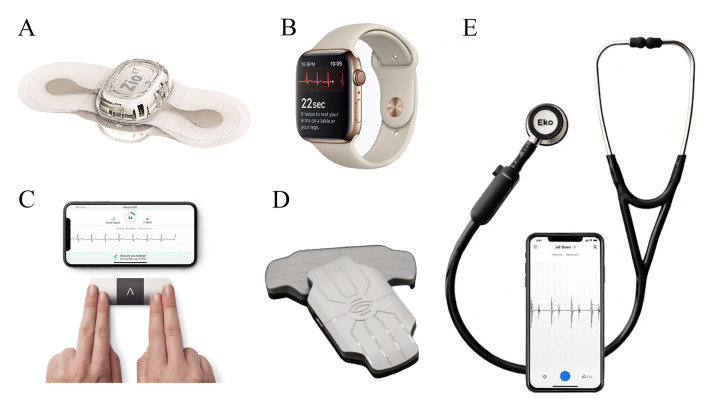
**Electrophysiological signal monitoring devices for heart failure (HF)**. (A) The Zio® XT Patch features a discreet, bandage-sized oval design, which is adhesively secured on the upper left chest. Its waterproof casing enables continuous ambulatory monitoring for up to 14 days. (B) Optical heart sensor of photoplethysmography (PPG) in the Apple Watch, located on the rear case, central to the skin-contact surface, employs light-emitting diodes (LEDs) to project light onto the subcutaneous vasculature. Photodiodes then detect the backscattered light, which is modulated by blood flow pulsations, enabling heart rate calculation. (C) KardiaMobile 6L Max™ device employs a three-point contact method: the user’s hands contact the two electrodes at each end of the device, establishing Leads I, II, and III. The back electrode contacts the left leg or chest (e.g., parasternal area) to form the Wilson’s central terminal, from which the augmented limb leads (aVR, aVL, aVF) are derived algorithmically. (D) “Wenxin Xiaoyi” Smart Patch features a miniaturized design with dimensions of 6 cm × 3 cm × 1 cm and a weight of approximately 13 g. Its ergonomic structure, fabricated from medical-grade materials, ensures secure skin contact and user comfort during prolonged monitoring. (E) Building upon the conventional stethoscope design, Eko CORE 500™ Digital Stethoscope incorporates three electrodes embedded within the diaphragm for ECG signal acquisition, enabling real-time waveform display and data transmission directly on the device. 40× amplification and active noise cancellation can be achieved, according to the manufacturer.

Recent advancements in ECG technology are progressing along two trajectories: Increasing public demand for prolonged, user-friendly, out-of-hospital monitoring has spurred the development of devices with enhanced wearability and simplicity. Innovations include reduced lead configurations and wireless dry-electrode patch systems (e.g., Zio® XT Patch), which improve patient compliance and comfort [74,75]. In addition, for high-risk HF populations requiring increased accuracy and reliability, multi-lead ECG devices (e.g., KardiaMobile 6L™) are being refined. Many now incorporate complementary sensing modalities such as photoplethysmography (PPG) and phonocardiography (PCG), exemplified by integrated solutions in consumer wearables, including the Apple Watch, enabling a more holistic appraisal of cardiac physiological status. These parallel developments are collectively paving the way toward highly individualized monitoring strategies tailored to the needs of diverse patient groups [76,77] (Fig. 3) (Table 4, Ref. [78,79,80,81,82,83,84,85]).

**Table 4.** **Representative intelligent monitoring devices for heart failure (HF) based on electrophysiological signal assessment**.

Device	Key features	Clinical evidence			Limitations
		Evidence level	Key studies	Outcomes	
Zio® Patch/Zio monitor	Disposable adhesive ECG monitor; enables continuous monitoring for up to 14 days, optimized for intermittent arrhythmia detection.	Randomized controlled trials; systematic reviews; large-scale real-world analyses	Barrett et al., 2014 [78]; Eysenck et al., 2020 [80]; Battisti et al., 2026 [79]	Higher arrhythmia detection yield compared with 24–48 h Holter monitoring [78,79]; AF burden detection comparable to implantable pacemakers [80].	Limited monitoring duration (≤14 days); single-lead configuration restricts accurate classification of atrial flutter and complex tachyarrhythmias; absence of real-time alerts.
Apple Watch	Smartwatch with integrated single-lead ECG; non-invasive, convenient daily monitoring; 30-second acquisition via crown contact; AF notification function supported.	Large prospective studies	Attia et al., 2022 [81]	Demonstrated feasibility for AF screening; AI-enabled ECG analysis showed potential for identifying left ventricular ejection fraction ≤40% [81].	Intended for screening rather than definitive diagnosis; single-lead ECG associated with false-positive and false-negative results; limited capability for non-AF arrhythmias.
KardiaMobile 6L™	Portable six-lead ECG device; 30-second measurement; Bluetooth connection with smartphone app; detects AF, bradycardia, tachycardia, ectopy; some models support QT interval measurement.	Prospective validation studies	EVALECG Cardio study; AI-LVSD study	High concordance with standard 12-lead ECG for rhythm diagnosis and QT/QTc interval assessment [82]; superior AF detection compared with single-lead smartwatch ECGs [83].	Requires active patient participation; no continuous automatic monitoring; dependent on high patient adherence; physician interpretation of ECGs remains necessary.
Eko CORE 500™ Digital Stethoscope	Digital stethoscope with integrated 3-lead ECG; AI-assisted analysis; noise reduction and heart sound amplification; real-time HR and ECG waveform display.	Multicenter prospective observational studies	TRICORDER study (NCT05987670)	Demonstrated feasibility of AI-assisted ECG and phonocardiogram analysis for detection of HF, AF, and valvular heart disease in real-world clinical settings [84,85].	Primarily intended for professional use, not consumer-grade; single-lead ECG limits diagnostic accuracy; heart sound analysis is susceptible to noise and probe placement; lacks extensive multicenter clinical validation.
“Wenxin Xiaoyi” Smart Patch	At-home patch device; simultaneous PCG and ECG recording; integrated analysis for arrhythmias, valve function, and early signs of HF.	Peer-reviewed clinical data are still relatively limited.			Single-lead ECG constrains accuracy; heart sound analysis is affected by ambient noise and sensor placement; as a newer device, large-scale multicenter clinical validations are still lacking.

AF, atrial fibrillation; LVSD, left ventricular systolic dysfunction; TRICORDER study, Triple Cardiovascular Disease Detection; AI, artificial intelligence.

Zio® XT Patch: It is a single-lead, long-term continuous ECG monitoring system designed as a lightweight, adhesive-based wearable device. It enables uninterrupted recording for up to 14 days, significantly extending the surveillance window compared to conventional 24–48 h Holter monitors. This extended duration is critical for capturing intermittent or subclinical arrhythmia, particularly in symptomatic patients without overt electrophysiological abnormalities during short-term monitoring [86]. Additionally, the Zio® XT integrates cloud-based data transmission and AI-driven analysis (ZEUS; iRhythm Technologies, Inc., San Francisco, CA, USA), facilitating remote clinician review and reducing reliance on in-person follow-ups.

Multiple clinical studies have demonstrated that extended monitoring with Zio significantly improves arrhythmia detection compared with conventional 24–48 h Holter monitoring. In a pivotal comparative study involving 146 patients, the Zio Patch identified 57% more clinically relevant arrhythmic events than standard Holter monitoring, largely due to prolonged wear duration [78]. A randomized trial comparing external ECG monitors further showed that AF burden detected by Zio was comparable to that recorded by implanted pacemakers, supporting its diagnostic reliability for AF surveillance [80]. Real-world analyses from 1,100,337 devices demonstrated that Zio XT monitoring was associated with higher diagnostic yield [79]. Although outcome-driven HF trials are lacking, its robust arrhythmia detection capability provides clinically meaningful information for HF risk stratification and management.

Apple Watch: The Apple Watch is a representative of smart wearables. Leveraging photoplethysmography (PPG), it utilizes green and infrared LEDs coupled with photodetectors to measure capillary blood volume changes, enabling non-invasive estimation of arterial compliance, pulse wave velocity (PWV), and HF hemodynamic burden. This technology facilitates continuous, portable monitoring of cardiovascular parameters, bridging the gap between consumer convenience and clinical utility [87,88].

Despite its classification as a consumer device, the Apple Watch has achieved FDA clearance for medically validated features, including single-lead electrocardiogram (ECG) recording, irregular rhythm notifications, and AF burden history. With multiparametric capabilities and low cost, it shows substantial potential in HF hemodynamic monitoring. According to Counterpoint Research, Apple led the global smartwatch market with a 20% share in Q1 2025, while the overall market valuation reached USD 5.057 billion (DemandSage data), highlighting ongoing growth in this field [89,90].

Large-scale prospective studies have validated its accuracy in detecting AF through irregular rhythm notifications and ECG recordings, leading to FDA clearance for AF screening. Beyond arrhythmia detection, a landmark prospective study demonstrated that AI-enabled analysis of Apple Watch single-lead ECGs could identify left ventricular systolic dysfunction (LVEF ≤40%) with good diagnostic performance (AUROC ~ 0.88–0.89), suggesting potential utility in HF screening [81]. However, existing evidence primarily supports screening and risk identification rather than definitive diagnosis or outcome modification. No randomized trials have yet shown that Apple Watch–guided monitoring reduces HF hospitalizations or mortality, highlighting the need for dedicated HF outcome studies.

KardiaMobile 6L™: The device provides six-lead ECG (I, II, III, aVL, aVR, aVF), offering a more comprehensive rhythm assessment compared to single-lead devices. Its compact design incorporates two finger electrodes and a leg-contact electrode, eliminating the need for adhesive patches or gels while enabling rapid 30-second ECG acquisition via Bluetooth-connected smartphones. The device transmits data to the KardiaCare™ subscription platform, which offers automated AI analysis (FDA-cleared Kardia AI algorithm) and optional review by certified cardiologists, supporting RM and timely intervention [91].

A large prospective validation study involving 1015 unselected cardiology patients showed strong correlation between 6-lead recordings and standard 12-lead ECGs across key parameters such as PR and QRS intervals, with AUC values >80%, supporting its diagnostic reliability [82]. Head-to-head comparisons have demonstrated that six-lead ECG devices achieve higher sensitivity and specificity for AF detection compared with single-lead smartwatches when interpreted by cardiologists (sensitivity ~99%, specificity ~97%) [83]. In addition, an AI-augmented six-lead ECG model based on KardiaMobile 6L recordings demonstrated high accuracy (AUROC ~0.92) for detecting left ventricular systolic dysfunction in a large prospective cohort, suggesting potential beyond simple rhythm classification [92].

Wenxin Xiaoyi Smart Heart Patch: It is the first portable device in China approved for simultaneous phonocardiography and ECG collection. Combining single-lead ECG with phonocardiographic analysis, it provides a more comprehensive evaluation of cardiac rhythm, valve closure, and structural abnormalities such as myocardial hypertrophy [93].

Eko CORE 500™ Digital Stethoscope: The Eko CORE 500™ represents the advancement in cardiac diagnostics as the first device to seamlessly integrate high-fidelity auscultation, 3-lead ECG, and AI-powered analysis into a single handheld tool. Its patented TrueSound™ technology provides 40× sound amplification with active noise cancellation and three specialized filtering modes, enabling identification of LVEF ≤40% within 15 seconds during routine examination [94].

In a large multicenter observational study, AI-assisted analysis of combined ECG and phonocardiographic signals enabled detection of HF with reduced ejection fraction, defined by an LVEF ≤40%, with an AUROC of 0.85, sensitivity of 84.8%, and negative predictive value exceeding 97% [84].

Ongoing pragmatic trials, including the TRICORDER study (NCT05987670), aim to evaluate real-world clinical implementation; however, randomized outcome trials assessing reductions in HF hospitalization or mortality are not yet available [85].

### 2.4 Intelligent Data Processing Techniques

**Fig. 4.**
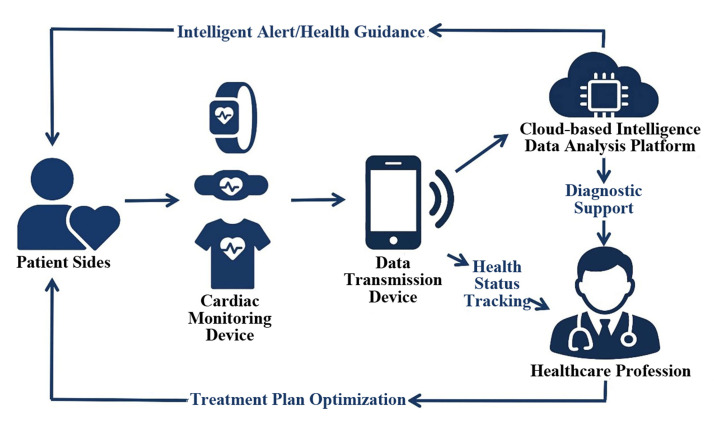
**Schematic of an intelligent remote monitoring (RM) platform for heart failure (HF) management**. The system operates via a continuous cycle: patients utilize wearable devices (e.g., smartwatches, specialized textiles) to collect cardiac data, which is transmitted via mobile networks to a cloud-based AI analytics platform. This platform processes multi-source medical data using machine learning (ML) to generate diagnostic support, issue intelligent alerts, and provide health guidance to clinicians, enabling optimized treatment plan adjustments and forming a closed-loop, patient-centric management ecosystem.

AI is a branch of computer science that involves mathematical algorithms designed to automate intelligent behaviors. In the medical field, AI demonstrates unique advantages in disease diagnosis and prognosis, especially for HF, a condition involving multi-system, multi-parameter, and multi-subtype assessments, due to its powerful adaptability, scalability, automation, and ability to process multidimensional and multivariable data [95,96,97]. Based on this, non-invasive intelligent management platforms for HF have gradually developed. These platforms are built on RM systems and rely on multi-source medical big data, utilizing AI machine learning (ML) as the core technology to achieve accurate modeling (Fig. 4). Currently, non-invasive intelligent management for HF has shown positive effects in early screening, exploring new care approaches, optimizing personalized treatment, and controlling disease progression [8,98,99].

#### 2.4.1 Development Status of Intelligent Remote Monitoring Platform for Heart Failure

Machine learning, a computational discipline, focuses on developing algorithmic models capable of identifying complex patterns and features within large-scale databases. Based on the type of input variables, ML can be categorized into supervised learning, semi-supervised learning, unsupervised learning, and reinforcement learning. These methodologies are selectively employed to address clinical and research challenges, such as the prediction of the individualized risk for HF, HF subtype classification, and just-in-time adaptive intervention (JITAI) [95].

**Fig. 5.**
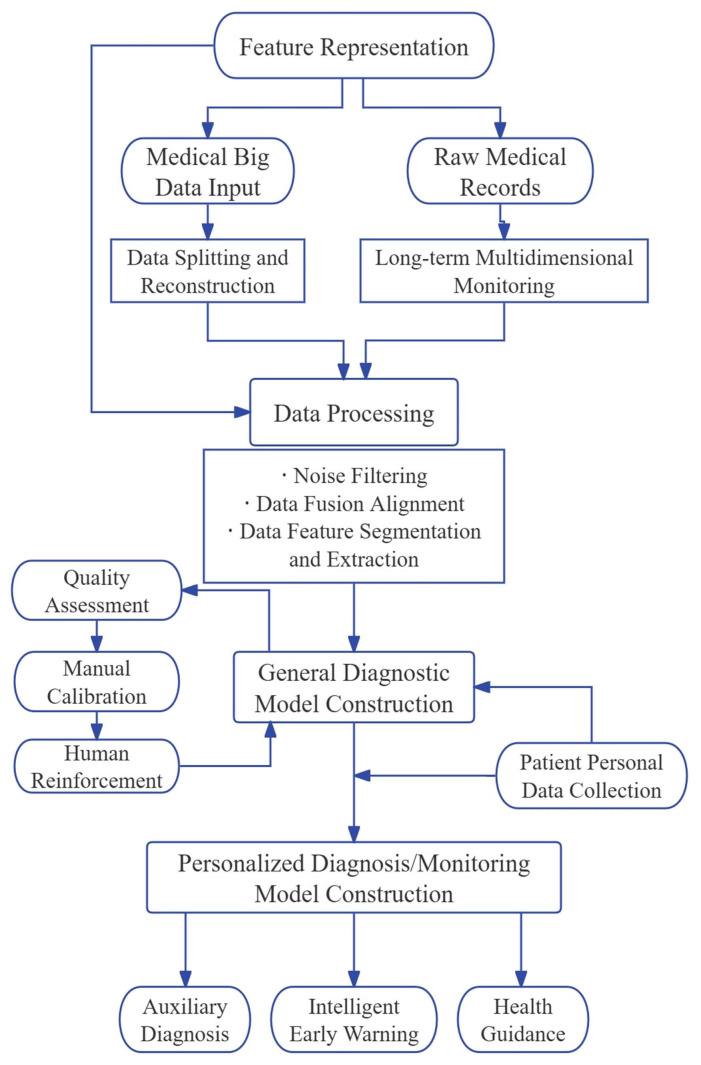
**Workflow for constructing an intelligent heart failure (HF) detection platform**. The process begins with feature representation from multi-source medical data, including electrophysiological, hemodynamic, and fluid status parameters. Following data preprocessing (noise filtering, fusion alignment, and feature extraction), a general diagnostic model is built and refined through quality assessment with human reinforcement. Integration of patient-specific data then enables personalized model development, supporting clinical applications in auxiliary diagnosis, early warning, and tailored health guidance.

Recent years have witnessed exponential growth in AI and ML applications within cardiovascular medicine, profoundly transforming HF diagnosis and prognosis management [100] (Table 5). The ML pathway is built on feature representation and involves the reorganization, integration, and alignment of original monitoring data from large datasets, including cardiac electrophysiology, hemodynamics, and tissue fluid status from medical records. Additionally, it incorporates active noise filtering and feature segmentation extraction for various signals to construct a generalized diagnostic model. Based on this model, personalized diagnostic models are further refined by incorporating individual-specific characteristics to maximize their performance [97,101,102] (Fig. 5).

**Table 5.** **Common intelligent detection platforms for heart failure (HF) available on the market**.

Platform name (company)	Platform description
HeartLogic™ (Boston Scientific, Marlborough, MA, USA)	A machine learning-based model calculates the HeartLogic™ index from five sensor-derived parameters (heart sounds, thoracic impedance, respiration, nighttime heart rate, and activity). When the index exceeds a preset threshold, the system issues an alert, indicating a potential risk of HF decompensation in the coming weeks.
CardioSignal (Precordior, Turku, Finland)	Utilizes smartphone-based gyroscope and accelerometer to detect specific rotational movements of the heart as raw data. The platform performs six-channel analysis to generate a gyrocardiogram and provides diagnostic and therapeutic recommendations, particularly suitable for early intervention in HF.
AliveCor KardiaMobile™ (AliveCor, Mountain View, CA, USA)	Built on a patented portable real-time ECG device. The device connects with a cloud platform, where AI algorithms analyze the data to detect possible signs of HF as well as other cardiac abnormalities.
CardioMEMS™ HF System (Abbott, Abbott Park, IL, USA)	Although CardioMEMS™ is an invasive implantable system, it is included in this table as a benchmark for intelligent remote monitoring platforms, providing a crucial reference for evaluating the clinical value of emerging non-invasive technologies. It continuously monitors pulmonary artery pressure and transmits data wirelessly to the medical team, helping assess the severity of HF and treatment effectiveness. The system uses data analysis algorithms to support clinical decision-making, reduce hospitalization and mortality rates, and improve the quality of life in patients with HFrEF and HFpEF.
Biotronik Home Monitoring® (Biotronik, Berlin, Germany)	Uses implantable devices (e.g., pacemakers, defibrillators, cardiac resynchronization therapy devices) to collect real-time cardiac health data, which are analyzed via an intelligent platform to provide early warnings of HF. It enables continuous care and early detection for timely intervention, reduces patient mortality, and can safely and effectively decrease or replace in-person follow-ups.

HFrEF, heart failure with reduced ejection fraction; HFpEF, heart failure with preserved ejection fraction.

From a clinical perspective, interpretability of these models is increasingly enhanced through feature attribution or attention-based approaches, which highlight the relative importance of input signals such as heart rate variability, impedance trends, or acoustic features. Post hoc explanation tools, including SHAP or LIME, have also been applied to complex models to provide case-level insights without altering the underlying algorithmic structure.

#### 2.4.2 Multi-scenario Applications of Intelligent Platforms

##### 2.4.2.1 Risk Prediction and Prognosis Management

For long-term users of health management platforms, intelligent HF monitoring systems can continuously analyze HF-related physiological parameters such as ECG and heart sounds, combined with the users’ electronic health records (EHRs), to promptly detect and report abnormalities. This significantly improves the early risk prediction of HF and the rate of detection of asymptomatic patients. A Chinese research team has demonstrated that independent factors such as age, regional poverty-to-income ratio, history of myocardial infarction, history of coronary artery disease, chest pain symptoms, and use of hypoglycemic agents can be used to predict the risk of HF among middle-aged and elderly patients with diabetes or prediabetes [103].

For patients already diagnosed and receiving treatment, this technology enables real-time tracking of cardiac status, critical value alerts, and personalized lifestyle interventions, effectively reducing readmission and mortality rates while improving long-term outcomes. The LINK-HF study showed that ML algorithms achieved a sensitivity of 76–88% and a specificity of 85% in detecting HF readmission, comparable to implantable devices, with alerts generated 6.5 days earlier than rehospitalization [104]. In the field of remote cardiac rehabilitation (CR), wearable device–based intelligent platforms have been shown to be as effective as center-based CR in improving physical activity (PA) and quality of life (QOL), while being cost-effective [105,106]. An umbrella review further indicated that intelligent platforms connected with wearable activity trackers can sustainably increase individuals’ daily PA by enhancing lifestyle habits and health awareness, thereby improving health status. This effect can be further enhanced through JITAI, offering promising opportunities for HF management [107,108,109].

##### 2.4.2.2 Assisted Diagnosis and Individualized Treatment Planning

An umbrella review further indicated that intelligent platforms connected with wearable activity trackers can sustainably increase individuals’ daily PA by enhancing lifestyle habits and health awareness, thereby improving health status. This effect can be further enhanced through JITAI, offering promising opportunities for HF management [110]. Such classification algorithms not only provide binary predictions of normal vs. diseased states with corresponding probabilities but also streamline traditional workflows that rely on echocardiography, thereby directly improving diagnostic efficiency and accuracy.

Continuous recording and analysis of patients’ cardiac physiological indicators over time also helps support the formulation of personalized HF treatment strategies. Experimental studies have already demonstrated the potential of intelligent platforms in multiple domains, including individualized transfusion decisions during treatment, prediction of heterogeneous responses to spironolactone in HFpEF patients, and surgical planning for ischemic HF patients [111,112,113,114]. Furthermore, the diversity of platform algorithms allows users to select and combine the most suitable models according to their health status and monitoring needs, optimizing user experience [115].

##### 2.4.2.3 Iterative Optimization of Diagnostic Criteria

Although the development of ML pathways is based on current clinical diagnostic standards, ML can also help overcome the limitations of traditional guidelines. Supervised learning shows strong potential in quantitatively supplementing existing HF diagnostic standards and building more accurate risk stratification models. Unsupervised learning, on the other hand, helps uncover hidden patient subtypes (e.g., distinct pathophysiological characteristics or progression patterns), thereby driving refinement and innovation in diagnostic frameworks [116].

Studies have shown that in predicting mortality or readmission risk during the vulnerable phase (within three months post-discharge) of acute HF patients, the XGBoost model outperformed traditional logistic regression models. Notably, key predictors in this model included serum uric acid and D-dimer levels, whose contributions even exceeded that of BNP, a commonly used HF biomarker, suggesting their potentially greater biological and clinical significance in risk stratification [117].

##### 2.4.2.4 Acceleration of Clinical Research Progress

Non-invasive intelligent platforms, through continuous data collection and real-time analysis, can significantly enhance clinical trial efficiency. On one hand, they enable precise participant screening and dynamic efficacy evaluation, thereby shortening trial cycles. On the other hand, remote monitoring and AI-assisted diagnostics reduce invasive risks and improve data reliability. In addition, individualized data allow for stratified efficacy assessment and evaluation of differential treatment responses, promoting the development of personalized therapeutic strategies. Automated endpoint assessment and virtual follow-up models further optimize resource allocation and reduce the operational costs of HF trials [118].

##### 2.4.2.5 Promoting the Development of Nationwide Health Management Systems

By integrating large-scale user EHR data via cloud platforms, intelligent systems can generate epidemiological landscape maps of HF (e.g., regional incidence, distribution of risk factors, treatment gaps), providing predictive insights and decision support for health authorities. Research has shown that ML models can accurately estimate HF-related risks (AUC = 0.91). Based on these findings, governments can dynamically adjust disease prevention strategies, resource allocation, and policy guidance, thereby promoting the construction of a patient-centered nationwide health management ecosystem [119].

## 3. Conclusions

### 3.1 Summary

HF remains a major global health burden, requiring a paradigm shift from reactive treatment to proactive, continuous management. This review integrates advances in non-invasive sensing technologies and AI analytics that enable dynamic monitoring of neurohormonal, hemodynamic, and metabolic alterations. Compared with conventional echocardiography and biomarkers, which lack continuous monitoring capabilities, multimodal sensing (e.g., ECG, hemodynamic signals, fluid status) offers earlier risk identification and intervention. Intelligent algorithms can enhance diagnostic precision, personalize disease management, and optimize resource allocation. Representative devices, such as the CardioMEMS HF System and the Zio Patch, illustrate the clinical feasibility of real-time monitoring and stratification. Together with evolving AI-enabled stethoscopes and wearable sensors, these tools represent a transition toward patient-centered, data-driven care. While significant challenges remain, particularly in accuracy, interoperability, and regulatory alignment, these technologies hold the potential to reshape HF management and pave the way toward precision cardiovascular medicine.

### 3.2 Limitations and Future Directions

#### 3.2.1 Improvement in Detection Device Sensitivity and Accuracy

Current intelligent HF detection platforms remain in the developmental phase. Limitations in sensor performance and analytical capabilities have led to high rates of misdiagnosis in clinical trials, with frequent false alarms disrupting the daily lives of patients. Therefore, the entire “sensor device–data calibration–data analysis” pipeline requires optimization, ensuring seamless compatibility and unified design.

Studies have shown that challenges persist due to confounding factors, such as obesity, pulmonary diseases, and skin tone, which affect data accuracy in commercial devices [120]. Additionally, biases in training data, such as racial or gender imbalances, may skew AI diagnoses, compromising clinical decisions [121]. Future efforts must focus on enhancing the sensitivity and accuracy of data collection instruments from multiple angles, including device design, technology upgrades, and material optimization. At the same time, strict verification of model fairness should be conducted, and more effective bias warning mechanisms, such as dynamic risk alerts and cross-validation using multiple models, should be developed to ensure user safety [122].

#### 3.2.2 Optimizing Long-Term Comfort and Adherence

Despite improved accessibility via wearables and patches, long-term adherence remains low, particularly among elderly or less tech-literate users. Research shows that over 30% of patients discontinue use after 3 months due to discomfort or cumbersome operation [123]. Future efforts should focus on materials science innovation to develop flexible, skin-friendly substrates through user-centric design, integrating unobtrusive monitoring and behavioral incentives to prolong engagement.

#### 3.2.3 Standardization of Multimodal Data and Improvement in Interoperability

Currently, the ECG, PPG, SCG, impedance, and imaging data collected by different platforms lack a unified standardized processing framework, making large-scale data integration across devices and centers difficult. Additionally, some intelligent platforms restrict database access due to commercial interests, further hindering data sharing.

In the future, a unified data format and labeling specification should be established based on internationally recognized standards (e.g., HL7 FHIR, IEEE 11073), integrating data from different hospitals and devices into continuous, comparable large-scale databases. This will not only facilitate multicenter collaborative trials but also enable compliant sharing and reuse in public health fields, such as the European Health Data Space (EHDS) framework [124,125,126].

#### 3.2.4 Real-Time Closed-Loop Monitoring and Intervention

Current HF systems suffer from delayed analysis due to centralized processing, limiting acute event response. A paradigm shift toward “monitoring-intervention-feedback” closed-loop systems is critical [5].

The key strategy lies in integrating advanced multi-parameter monitoring technologies with real-time treatment devices. It will not only drive the paradigm shift from “passive treatment” to “active intervention” but also significantly improve patient outcomes through automated closed-loop responses.

#### 3.2.5 Mitigating the AI “Black Box” Dilemma

The opacity of AI decision-making undermines trust in HF platforms. The “black box” problem refers to the phenomenon where, due to the high complexity of deep learning models, users can observe the input and output of AI systems but cannot understand the internal decision-making logic or reasoning process. Although models such as linear/logistic regression and decision trees are inherently interpretable, and tools like SHAP/LIME have improved the post-interpretability of complex models, gaps persist in explaining risk factors or treatment logic. This significantly reduces patient and healthcare professional trust in the platform’s output, potentially creating difficulties in patient adherence and the clarification of medical responsibility during subsequent treatments [127,128,129].

Future HF detection platforms must adhere to good machine learning practices (GMLP) and the “transparency principle”, developing standardized metrics for evaluation and providing clinical teams with actionable explanations, such as identifying specific risk factors and possible intervention options [130]. Moreover, graded responses and simplified alerts should be used to reduce “alarm fatigue” and improve physician acceptance.

#### 3.2.6 Ensuring Data Privacy and Regulatory Compliance

AI diagnostic models rely on big data platforms, requiring users to upload personal health information to the cloud for processing, which raises widespread concerns about data privacy. Despite ongoing efforts to improve regulations in various countries, such as the U.S. Health Insurance Portability and Accountability Act (HIPAA) and the European General Data Protection Regulation (GDPR), surveys indicate that more than half of the public still doubts the adequacy of these regulations in terms of completeness, enforcement, and scope [97,131].

Future efforts should focus on developing secure, compliant cross-hospital and cross-border data collaboration processes. This will include using differential privacy technology to protect personal data, implementing multi-party secure computing for joint modeling across institutions, and establishing standardized black-box/white-box simulation mechanisms to test system resilience against attacks. A full-chain security framework encompassing “risk modeling—protection—compliance auditing” will be promoted.

#### 3.2.7 Navigating Insurance and Regulatory Pathways

Although AI has been widely applied in many fields, its deep integration into healthcare, particularly in HF monitoring, remains in its early stages. The regulatory frameworks governing AI-enabled devices and software as a medical device (SaMD) vary substantially across regions and require clear evidence of clinical safety and effectiveness. The pathway from clinical validation to consistent insurance coverage is still evolving, and many health systems have not established stable reimbursement structures for novel RM technologies and AI algorithms. Policy-driven initiatives over the next decade will play a decisive role in shaping the adoption of these technologies. In particular, encouraging countries with rapidly aging populations, such as China, to use insurance payments and tiered healthcare combined with smart healthcare will facilitate its implementation and benefit a large HF patient population.

#### 3.2.8 Coverage and Reimbursement

Building on the discussion of regulatory pathways, a major practical barrier to the broad implementation of remote HF monitoring lies in insufficient coverage and limited reimbursement for both implantable and ambulatory monitoring technologies. Surveys and dedicated reports illustrate that in many countries there is inadequate payer support for cardiac implantable electronic devices (CIEDs), including implantable loop recorders (ILRs), and for ambulatory monitoring devices such as patches and extended surveillance systems [132,133,134]. Across Europe, access and reimbursement for ambulatory cardiac monitoring and ILRs exhibit large inter-country variability, with monetary reimbursement values ranging widely and newer long-duration ambulatory technologies often lacking coverage altogether, despite evidence of their clinical utility (e.g., significant variance in Holter and ILR reimbursement levels).

Importantly, reimbursement practices continue to be heterogeneous even within regions where RM is recognized as standard follow-up care for CIEDs. Recent analyses highlight that inconsistent payer policies and a lack of well-defined reimbursement codes remain among the primary barriers to the full implementation of RM into standard practice, thereby slowing its diffusion and potentially limiting patient access to guideline-based follow-up care [135]. Addressing these barriers is crucial for several reasons.
